# Mitochondrial mass and activity as a function of body composition in individuals with spinal cord injury

**DOI:** 10.14814/phy2.13080

**Published:** 2017-02-13

**Authors:** Laura C. O'Brien, Rodney C. Wade, Liron Segal, Qun Chen, Jeannie Savas, Edward J. Lesnefsky, Ashraf S. Gorgey

**Affiliations:** ^1^ Spinal Cord Injury and DisordersHunter Holmes McGuire VA Medical CenterRichmondVirginia; ^2^ Department of Physiology and BiophysicsVirginia Commonwealth UniversityRichmondVirginia; ^3^ Department of MedicineDivision of CardiologyPauley Heart CenterVirginia Commonwealth UniversityRichmondVirginia; ^4^ Department of SurgeryHunter Holmes McGuire VA Medical CenterRichmondVirginia; ^5^ Department of SurgeryVirginia Commonwealth UniversityRichmondVirginia; ^6^ Medical ServiceHunter Holmes McGuire VA Medical CenterRichmondVirginia; ^7^ Department of Biochemistry and Molecular BiologyVirginia Commonwealth UniversityRichmondVirginia; ^8^ Physical Medicine and Rehabilitation, Virginia Commonwealth UniversityRichmondVirginia

**Keywords:** Body composition, metabolism, Mitochondria, skeletal muscle, spinal cord injuries

## Abstract

Spinal cord injury (SCI) is accompanied by deterioration in body composition and severe muscle atrophy. These changes put individuals at risk for insulin resistance, type II diabetes, and cardiovascular disease. To determine the relationships between skeletal muscle mitochondrial mass, activity, and body composition, 22 men with motor complete SCI were studied. Body composition assessment was performed using dual‐energy X‐ray absorptiometry and magnetic resonance imaging. Skeletal muscle biopsies were obtained from the vastus lateralis muscle to measure citrate synthase (CS) and complex III (CIII) activity. CS activity was inversely related to %body fat (*r* = −0.57, *P* = 0.013), %leg fat (*r* = −0.52, *P* = 0.027), %trunk fat (*r* = −0.54, *P* = 0.020), and %android fat (*r* = −0.54, *P* = 0.017). CIII activity was negatively related to %body fat (*r* = −0.58, *P* = 0.022) and %leg fat (*r* = −0.54, *P* = 0.037). Increased visceral adipose tissue was associated with decreased CS and CIII activity (*r* = −0.66, *P* = 0.004; *r* = −0.60, *P* = 0.022). Thigh intramuscular fat was also inversely related to both CS and CIII activity (*r* = −0.56, *P* = 0.026; *r* = −0.60, *P* = 0.024). Conversely, lean mass (*r* = 0.75, *P* = 0.0003; *r* = 0.65, *P* = 0.008) and thigh muscle cross‐sectional area (CSA;* r* = 0.82, *P* = 0.0001; *r* = 0.84; *P* = 0.0001) were positively related to mitochondrial parameters. When normalized to thigh muscle CSA, many body composition measurements remained related to CS and CIII activity, suggesting that %fat and lean mass may predict mitochondrial mass and activity independent of muscle size. Finally, individuals with SCI over age 40 had decreased CS and CIII activity (*P* = 0.009; *P* = 0.004), suggesting a decrease in mitochondrial health with advanced age. Collectively, these findings suggest that an increase in adipose tissue and decrease in lean mass results in decreased skeletal muscle mitochondrial activity in individuals with chronic SCI.

## Introduction

Obesity, type II diabetes mellitus, metabolic syndrome, and cardiovascular disease are disorders that increase at an alarming rate in persons with spinal cord injury (SCI). The aforementioned comorbidities are preceded by dramatic changes in body composition and metabolic profile (Gorgey et al. 2014; Gorgey and Dudley 2007). Detrimental skeletal muscle atrophy and fiber type conversion from oxidative to fast glycolytic are likely to occur within the first year of injury (Castro et al. 1999; Gorgey and Dudley 2007). This results in a muscle that is highly fatigable and susceptible to exercise‐induced muscle damage. Moreover, whole body and regional (trunk and leg) lean mass are even lower when compared with matched able‐bodied controls (Monroe et al. 1998; Spungen et al. 2003). The loss of metabolically active muscle mass contributes to decreased basal metabolic rate and may lead to obesity. There is also an increase in total body fat mass and percentage fat per unit body mass index (BMI) that was observed in monozygotic twins with paraplegia compared to their able‐bodied twins (Spungen et al. 2000). Within the SCI population, age and level of injury affect body composition (Spungen et al. 2003). Individuals aged 40 or above had less % lean mass and more % fat mass than younger individuals (Spungen et al. 2003). Additionally, tetraplegics were found to have less lean body mass than paraplegics (Spungen et al. 2003).

Sublesional deterioration in lean mass and muscle quality predisposes this population to remarkable ectopic adipose tissue accumulation. This is characterized by infiltration of intramuscular fat (IMF) and visceral adipose tissue (VAT). Infiltration of IMF is observed only 6 weeks after SCI (Gorgey and Dudley 2007) and is negatively associated with glucose tolerance following oral glucose challenges in individuals with chronic SCI (Elder et al. 2004). Moreover, the central adiposity observed after SCI is associated with altered metabolic profile and is a risk factor for cardiovascular and metabolic diseases (Gorgey et al. 2014, 2011; Gorgey and Gater 2011a; Jensen 2008). However, it remains unclear how the above changes in body composition after SCI are likely to impact cellular function and trigger medical comorbidities.

Recent evidence suggests that mitochondrial activity is impaired in metabolic disorders such as obesity, type II diabetes mellitus, metabolic syndrome, and cardiovascular disease (Chan 2006; Phielix and Mensink 2008). Skeletal muscle mitochondria are smaller and less active in obese and type II diabetics compared to healthy controls (Kelley et al. 2002; Ritov et al. 2010). Similarly, decreased mitochondrial gene expression, decreased mitochondrial DNA content, and increased mitochondrial DNA deletions are seen in skeletal muscle from aged individuals (Carter et al. 2015; Cooper et al. 1992). Decreased function of the electron transport chain (ETC) is also observed with age, with declines in mitochondrial respiration and maximum ATP production rates (Cooper et al. 1992; Joseph et al. 2012). This may result in impaired glucose utilization and decreased daily energy expenditure. It remains unknown what factors influence this decline in mitochondrial activity and what interventions are needed to reverse this process.

Little is known about mitochondrial activity after SCI. Previous studies using indirect measurements of mitochondrial activity such as near‐infrared spectroscopy and ^31^P magnetic resonance spectroscopy suggest that skeletal muscle oxidative capacity is impaired by 50–60% after SCI (Erickson et al. 2013; McCully et al. 2011). Histological analysis revealed a decrease in succinate dehydrogenase activity, complex II of the ETC, in skeletal muscle of individuals with chronic SCI (Grimby et al. 1976; Martin et al. 1992; Rochester et al. 1995). The master regulator of mitochondrial biogenesis, peroxisome proliferator‐activated receptor gamma coactivator 1‐alpha (PGC‐1*α*), and its downstream targets were decreased following nerve denervation in rodents (Adhihetty et al. 2007). This coincided with decreased enzyme activity of complex IV, decreased respiration, and increased reactive oxygen species in the deinnervated muscle (Adhihetty et al. 2007). It remains unclear if a decrease in mitochondrial mass or a deficit in ETC activity may be related to changes in muscle size, ectopic adipose tissue accumulation, or total body composition. Establishing this relationship may provide insights on the importance of skeletal muscle mitochondrial mass and activity to overall health. Interventions that induce muscle hypertrophy may increase skeletal muscle oxidative function, increase daily energy expenditure, and reduce adiposity.

The aim of this study was to investigate the relationship between body composition and mitochondrial mass and activity in skeletal muscle biopsies from individuals with SCI. Because of the high density of mitochondria in skeletal muscle compared with other tissues, the hypothesis was that individuals with increased lean mass would have increased skeletal muscle mitochondrial mass and activity. Conversely, those with increased adipose tissue would have decreased mitochondrial enzyme activity. If confirmed, these findings may highlight the importance of increasing or maintaining lean mass and decreasing adipose tissue deposition after SCI. This may help guide the development of interventions in order to improve the health of individuals and prevent medical comorbidities following SCI. These findings may be applicable to many other clinical populations including cardiovascular disease, type II diabetes, insulin resistance, and obesity.

## Methods

### Ethical approval

All aspects of the study were reviewed and approved by the McGuire VA Medical Center institutional review board. This research was performed as part of a clinical trial, registered at clinicaltrials.gov (NCT01652040). Subjects provided written informed consent prior to enrollment in the study. Data presented in this study are cross‐sectional prior to conducting any intervention.

### Participants

Twenty‐two men between the ages of 18 and 50 with a BMI ≤31.5 kg/m^2^ were invited to participate in the study. Participants had motor complete SCI (American Spinal Injury Association (ASIA) impairment scale classification A (*n* = 16) or B (*n* = 6)) at least 1 year prior to the start of the study. Levels of injury ranged from T11 to C5. Subject demographics are shown in Table 1. None of the subjects had preexisting conditions including cardiovascular disease, pressure sores stage II or greater, or uncontrolled type II diabetes. After providing written informed consent, subjects underwent a complete physical examination by a board‐certified physiatrist including neurological assessment and ASIA examination.

**Table 1:** Subject demographics

** **	Tetra	Para	Total
Demographics (*n*)	8	14	22
Age, year	37.5 ± 11.6	35.3 ± 9.4	36.1 ± 10.0
Height, m	1.80 ± 0.05	1.78 ± 0.07	1.79 ± 0.06
Weight, kg	75 ± 14	80 ± 13	78 ± 13
BMI, kg/m^2^	23.3 ± 4.5	25.3 ± 3.4	24.6 ± 3.9
TSI, y	7.9 ± 7.2	8.4 ± 8.5	8.2 ± 7.9
Caucasian, *n*	6	8	14
African American, *n*	2	6	8

Values are means ± SD; *n*, number of subjects; Tetra, tetraplegics; Para, paraplegics; BMI, body mass index; TSI, time since injury

### Body composition

Weight was measured while subjects were seated in their wheelchairs, using a wheelchair scale (Tanita, Arlington Heights, IL). Once the subject was transferred to the mat to measure height, the weight of the wheelchair was measured empty and subtracted from the total weight. Height was measured in a supine position by placing a board at the soles of the feet and measuring height to the nearest 0.1 cm. Total body and regional dual‐energy X‐ray absorptiometry (DXA) scans were performed with a Lunar Prodigy Advance scanner (Lunar Inc., Madison, WI) after lower extremity elevation for at least 30 min. The trunk region included the neck, chest, abdominal, and pelvic areas. The android region was defined as the region between the ribs and the pelvis. Whole body %fat mass and lean mass was calculated after excluding bone tissue. The coefficient of variability in repeated DXA scans is less than 3%.

### Magnetic resonance image (MRI)

MRI images were obtained with a GE Signa 1.5 Tesla Magnet. Transaxial images, 8 mm thick and 16 mm apart, were taken from the hip joint to the knee joint for thigh analysis. For analysis of subcutaneous adipose tissue (SAT) and VAT, transverse slices (0.8 cm thick) were acquired every 0.4 cm from the xiphoid process to the femoral heads. Images were acquired in two stacks with L4–L5 as a separating point. Both legs were strapped with an elastic band to avoid movement due to muscle spasms. The subjects were asked to hold their breath to reduce breathing artifact. Analysis was performed using Win‐vessel software (Ronald Meyer, MSU) by an experimenter blinded to the experimental conditions as previously described (Gorgey and Dudley 2007; Gorgey et al. 2012). Briefly, images were segmented into fat, muscle, and bone based on signal intensity. VAT, SAT, whole thigh cross‐sectional area (CSA), and knee extensor CSA were measured by manually tracing around anatomical borders. The number of pixels in the highlighted region was multiplied by the matrix size to measure CSA (cm^2^). Absolute values are used for analysis and thigh data were taken for the right leg. MRI data were not available from one participant.

### Enzyme assays

Biopsy samples of vastus lateralis muscle were obtained by a 14 gauge tru‐cut^™^ biopsy needle, immediately frozen in liquid nitrogen, and stored at −70°C until analysis. A portion of this sample (~10–25 mg) was homogenized in 220 mmol/L mannitol, 70 mmol/L sucrose, 5 mmol/L MOPS, 2 mmol/L EDTA, with cOmplete^™^ protease inhibitor cocktail (Sigma‐Aldrich), pH 7.4. Samples were centrifuged at 2000 rpm (371*g*) for 5 min at 4°C and the supernatant was used for analysis. After protein concentration was determined by the Lowry method, samples were solubilized in 1% potassium cholate. Samples were analyzed on the same day as homogenization. Enzyme activity was measured spectrophotometrically at 37°C using a Hewlett‐Packard diode array spectrophotometer. Complex III (CIII) activity was determined as the antimycin A‐sensitive increase in absorbance at 550 nm for 45 sec, representing the reduction in cytochrome c coupled to the oxidation of ubiquinol to ubiquinone as previously described (*n* = 15) (Brass et al. 2001; Spinazzi et al. 2012). Seven samples were excluded from CIII analysis due to insufficient muscle tissue. Citrate synthase (CS), a marker of mitochondrial mass, was measured by the formation of the thionitrobenzoate anion at a wavelength of 412 nm for 90 sec after addition of 5,5‐dithiobis‐(2,4‐nitrobenzoic acid), acetyl‐CoA, and oxaloacetate (*n* = 18) as previously described (Brass et al. 2001; Spinazzi et al. 2012). Absorbance was measured before and after the addition of oxaloacetate and background absorbance was subtracted from the final reading. Four samples were excluded from CS analysis due to insufficient muscle tissue. Each sample was run in duplicate or triplicate for each assay, depending on the amount of sample available. Data were converted from arbitrary units per minute to nmol/min by using the extinction coefficients of 13.6 mM^−1 ^cm^−1^ for CS and 19.1 mM^−1 ^cm^−1^ for CIII. Data were normalized to mg of protein added.

### Statistics

Pearson's correlation coefficients and partial correlations (accounting for age or time since injury (TSI) as confounding variables) were used to identify associations between whole and regional body composition variables and CS and CIII activity. Independent *t*‐tests were used to determine the difference in body composition measurements and mitochondrial enzyme activity between groups based on age, level of injury, and TSI. All values are presented as means ± SD. Statistical analysis was performed using IBM‐SPSS version 23 (Armonk, NY).

## Results

### Subject characteristics

Subject demographics are shown in Table 1. Fourteen participants were paraplegic (T4–T11) and eight were tetraplegic (C5–C7). Participants ranged in age from 18 to 50 and BMI ranged from 17.1 to 31.5 kg/m^2^. Age, height, weight, BMI, and TSI were not statistically significant between tetraplegics and paraplegics or between Caucasians and African Americans. Body composition, visceral adiposity and thigh skeletal muscle, and mitochondrial enzyme measurements are described in Table 2. Values were not significantly different between paraplegics and tetraplegics. However, there was a trend for decreased lean mass (*P* = 0.09) and knee extensor CSA (*P* = 0.09) in tetraplegics.

**Table 2:** Subject characteristics

	Tetra	Para	Total
Body composition (*n*)	8	14	22
%Total Fat	35.40 ± 9.0	31.07 ± 10.1	32.6 ± 9.7
Total fat mass (kg)	27.81 ± 10.9	25.59 ± 10.2	26.40 ± 10.2
Total lean mass (kg)	45.83 ± 4.3	51.58 ± 8.3^a^	49.49 ± 7.5
%Leg fat	36.58 ± 9.2	32.74 ± 9.9	34.13 ± 9.6
Leg fat mass (kg)	8.37 ± 4.0	7.97 ± 3.4	8.11 ± 3.5
Leg lean mass (kg)	12.83 ± 2.7	14.99 ± 3.9	14.20 ± 3.6
%Trunk Fat	38.46 ± 9.7	35.19 ± 11.6	36.38 ± 10.8
Trunk fat mass (kg)	16.02 ± 6.7	14.47 ± 6.0	15.03 ± 6.1
Trunk lean mass (kg)	22.25 ± 5.2	22.56 ± 4.1	23.81 ± 3.3
%Android fat	42.25 ± 11.3	38.69 ± 12.7	39.99 ± 12.1
Android fat mass (kg)	2.98 ± 1.6	2.40 ± 1.1	2.61 ± 1.3
Android lean mass (kg)	3.68 ± 1.0	3.44 ± 0.5	3.53 ± 0.7
VAT and SAT (*n*)	8	13	21
SAT (cm^2^)	163.45 ± 103.2	152.41 ± 69.4	157.21 ± 79.6
VAT (cm^2^)	116.60 ± 56.3	89.91 ± 63.0	105.1 ± 63.6
VAT/SAT (cm^2^)
	0.83 ± 0.4	0.61 ± 0.4	0.73 ± 0.4
Thigh skeletal muscle (*n*)	8	12	20
%MF (thigh)	15.74 ± 6.7	12.99 ± 10.1	14.09 ± 8.8
Thigh muscle IMF (cm^2^)	16.06 ± 7.3	12.37 ± 9.0	13.92 ± 8.3
KE IMF (cm^2^)	4.56 ± 2.6	3.96 ± 4.4	4.20 ± 3.7
Thigh muscle CSA (cm^2^)	74.85 ± 16.5	87.16 ± 23.2	82.24 ± 21.2
KE CSA (cm^2^)
	32.72 ± 7.7	39.98 ± 11.3^a^	37.07 ± 10.4
Enzyme activity			
CS activity (nmol/mg/min)	40.4 ± 25.3, *n* = 7	60.6 ± 29.7, *n* = 11	54.0 ± 28.9, *n* = 18
CIII activity (nmol/mg/min)	25.0 ± 18.0, *n* = 6	40.3 ± 16.9, *n* = 9	41.6 ± 24.6, *n* = 15

Values are means ± SD; *n*, number of subjects; Tetra, tetraplegics; Para, paraplegics; VAT, visceral adipose tissue; SAT, subcutaneous adipose tissue; IMF, intramuscular fat; CSA, cross‐sectional area; CS, citrate synthase; CIII, complex III.

a *P* ≤ 0.1 tetra versus para.

**Figure 1:**
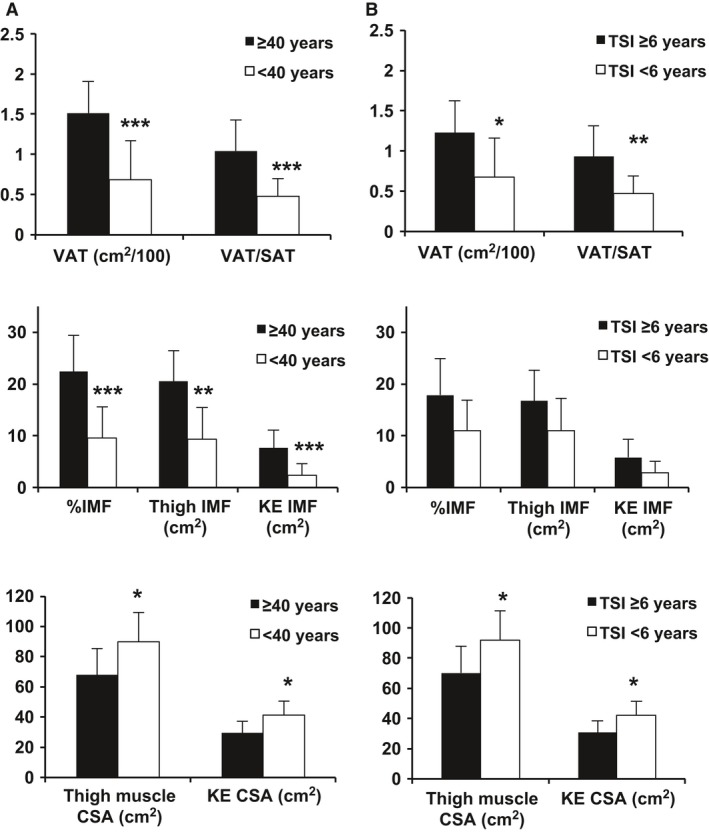
VAT, IMF, and muscle CSA comparisons between older (≥40, *n* = 8) and younger (<40, *n* = 13) individuals with SCI (A) and between TSI ≥6 (*n* = 10) and <6 (*n* = 11) (B). SAT, subcutaneous adipose tissue; KE, knee extensor; *P < 0.05, **P < 0.001, ***P < 0.0001. SCI, Spinal cord injury; TSI, time since injury; VAT, visceral adipose tissue; IMF, intramuscular fat; CSA, cross‐sectional area.

Figure 1 describes changes in VAT, IMF, and muscle CSA with age and TSI. There were no significant differences in body composition measurements measured by DXA between individuals age ≥40 (*n* = 8) and <40 (*n* = 13), or TSI ≥ 6 (*n* = 10) and <6 (*n* = 11) years (data not shown). However, there was a significant increase in VAT and VAT/SAT between individuals age ≥40 and <40 (*P* = 0.0007 and 0.0004, respectively) and between individuals with TSI ≥6 and <6 years (*P* = 0.04, *P* = 0.005, respectively). Older individuals also had increased %IMF, midthigh IMF, and knee extensor IMF (*P* = 0.0005, 0.001, and 0.0007, respectively). There was a trend for increased %IMF (*P* = 0.08) and KE IMF (*P* = 0.07) with TSI ≥6 years. Older individuals and those with longer TSI had decreased thigh muscle CSA (*P* = 0.02 and 0.02, respectively) and knee extensor CSA (*P* = 0.01 and 0.01, respectively).

### Relationships between mitochondrial mass, activity, and body composition

**Figure 2:**
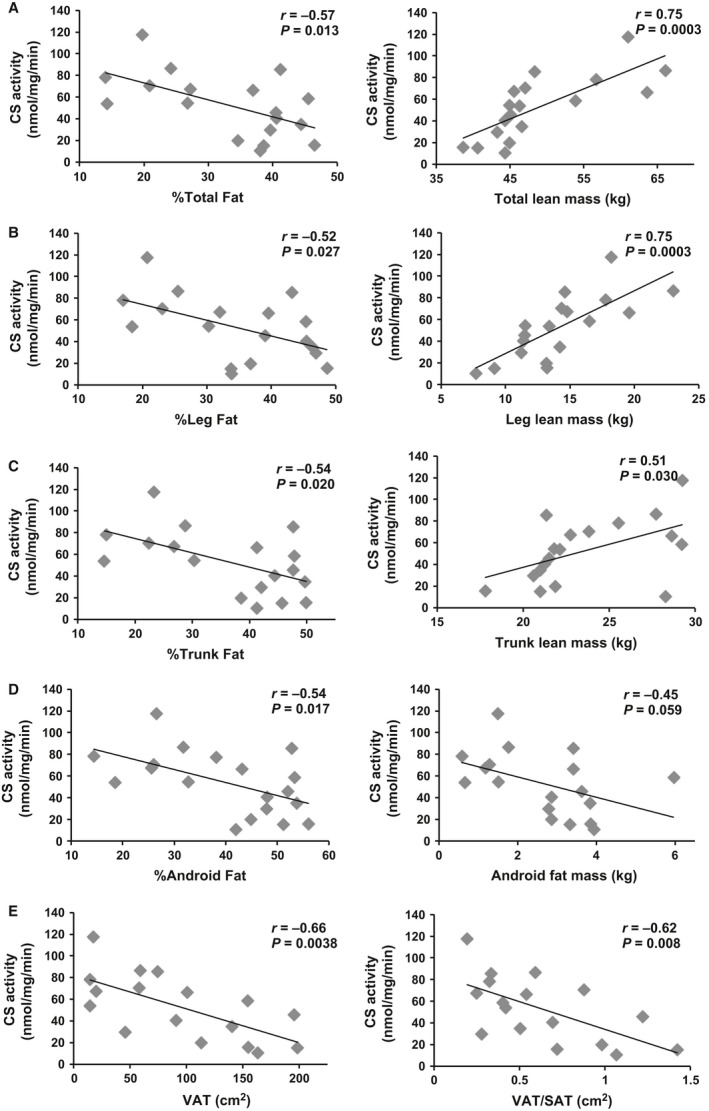
CS activity related to body composition measurements in total (A), leg (B), trunk (C), and android (D) compartments (*n* = 18). Visceral adiposity related to CS is shown in E (*n* = 17). VAT, visceral adipose tissue; SAT, subcutaneous adipose tissue; VAT, visceral adipose tissue.

**Figure 3:**
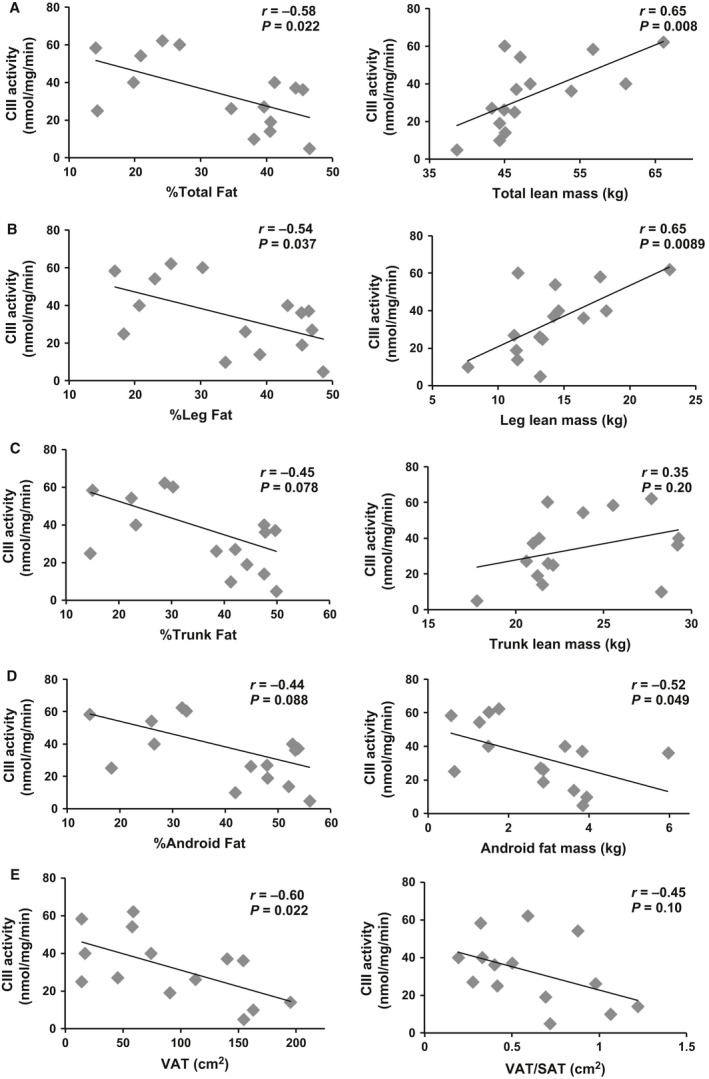
Antimycin A‐sensitive complex III (CIII) activity related to body composition measurements in total (A), leg (B), trunk (C), and android (D) compartments (*n* = 15). Visceral adiposity related to CIII activity is shown in E (*n* = 14). VAT, visceral adipose tissue; SAT, subcutaneous adipose tissue; CS, Citrate synthase.

The relationships between CS or CIII activity and body composition are shown in Figures 2 (*n* = 17–18) and Figure 3 (*n* = 14–15), respectively. Percent total body fat was inversely related to both CS activity (*r* = −0.57, *P* = 0.013) and CIII activity (*r* = −0.58, *P* = 0.022). These relationships were no longer significant when partial correlations were run to account for age (Table 3). However, when TSI was accounted for significant relationships remained (Table 3). In contrast, total lean mass was positively related to both CS and CIII activity (*r* = 0.75, *P* = 0.0003, and *r* = 0.65, *P* = 0.008, respectively). The relationship between total lean mass and CS but not CIII activity remained significant when age was accounted for (Table 3). When TSI was accounted for both relationships remained significant (Table 3). Total fat mass was not significantly related to CS or CIII activity (data not shown).

**Table 3:** Partial correlations accounting for TSI or age

	**CS**		**CIII**	
	*r*	*P*	*r*	*P*
%Total fat				
Pearson	**−0.57**	**0.013** *	**−0.58**	**0.022** *
Partial (TSI)	**−0.54**	0.025*	**−0.58**	**0.031** *
Partial (Age)	**−**0.37	0.143	**−**0.31	0.286
Total lean mass (kg)				
Pearson	**0.75**	**<0.001** **	**0.65**	**0.008** *
Partial (TSI)	**0.82**	**<0.001** **	**0.62**	**0.019** *
Partial (Age)	**0.79**	**<0.001** **	0.53	0.054^
%Leg fat				
Pearson	**−0.52**	0.027*	**−0.54**	**0.037** *
Partial (TSI)	**−0.55**	**0.022** *	**−0.57**	**0.033** *
Partial (Age)	**−**0.38	0.130	**−**0.35	0.218
Leg lean mass (kg)				
Pearson	**0.75**	<0.001**	**0.65**	**0.009** *
Partial (TSI)	**0.73**	**0.001** **	**0.57**	**0.033** *
Partial (Age)	**0.73**	0.001**	**0.53**	**0.049** *
%Trunk fat				
Pearson	**−0.54**	**0.020** *	**−**0.45	0.078^
Partial (TSI)	**−0.49**	**0.046** *	**−0.55**	**0.042** *
Partial (Age)	**−**0.30	0.240	**−**0.25	0.394
Trunk lean mass (kg)				
Pearson	**−0.51**	**0.030** *	0.35	0.200
Partial (TSI)	**0.68**	**0.003** *	0.47	0.091^
Partial (Age)	**0.59**	**0.013** *	0.32	0.258
%Android fat				
Pearson	**−0.54**	**0.017** *	**−**0.44	0.088^
Partial (TSI)	**−0.50**	**0.042** *	**−0.57**	**0.035** *
Partial (Age)	**−**0.30	0.242	**−**0.27	0.358
VAT (cm^2^)				
Pearson	**−0.66**	**0.004** *	**−0.60**	**0.022** *
Partial (TSI)	**−0.56**	**0.023** *	**−**0.54	0.056^
Partial (Age)	**−**0.43	0.097	**−**0.12	0.708
Thigh %IMF				
Pearson	**−0.56**	**0.026** *	**−0.60**	**0.024** *
Partial (TSI)	**−**0.41	0.132	**−0.59**	**0.034** *
Partial (Age)	**−**0.20	0.485	**−**0.30	0.319
Thigh muscle CSA (cm^2^)				
Pearson	**0.82**	**<0.001** **	**0.84**	**<0.001** **
Partial (TSI)	**0.71**	**0.003** *	**0.82**	**0.001** *
Partial (Age)	**0.75**	**0.001** *	**0.81**	**0.001** *
KE CSA (cm^2^)				
Pearson	**0.74**	**0.001** *	**0.84**	**<0.001** **
Partial (TSI)	**0.60**	**0.020** *	**0.82**	**0.001** *
Partial (Age)	**0.59**	**0.018** *	**0.75**	**0.003** *

CS, citrate synthase; CIII, complex III; TSI, time since injury; VAT, visceral adipose tissue; SAT, subcutaneous adipose tissue; IMF, intramuscular fat; KE, knee extensor; CSA, cross‐sectional area.

^^^ *P* < 0.1, *P < 0.05, **P < 0.001.

The relationship between body composition and skeletal muscle mitochondrial mass and activity in the leg, trunk, and android compartments were analyzed individually. Similar to total body, %leg fat (*r* = −0.52, *P* = 0.027), %trunk fat (*r* = −0.54, *P* = 0.020), and %android fat (*r* = −0.54, *P* = 0.017) were all negatively related to CS activity. Percent leg fat (*r* = −0.54, *P* = 0.037) was related to CIII activity with a trend for significance between %trunk fat and %android fat (*r* = −0.45, *P* = 0.078, and *r* = −0.44, *P* = 0.088, respectively). Android fat mass was negatively related to CIII (*r* = −0.52, *P* = 0.049) activity with a trend for significance when related to CS activity (*r* = −0.45, *P* = 0.059). When age was accounted for, these relationships were no longer statistically significant. However, when TSI was accounted for these relationships remained statistically significant (Table 3).

Leg (*r* = 0.75, *P* = 0.0003) and trunk (*r* = 0.51, *P* = 0.030) lean mass were both positively related to CS. These relationships remained statistically significant when corrected for age or TSI (Table 3). Leg lean mass (*r* = 0.65, *P* = 0.0089) was also related to CIII activity and this relationship remained when age or TSI was accounted for (Figure 2, Table 3). Trunk lean mass was not significantly related to CIII activity. Android lean mass was not significantly related to either parameter (data not shown).

**Figure 4:**
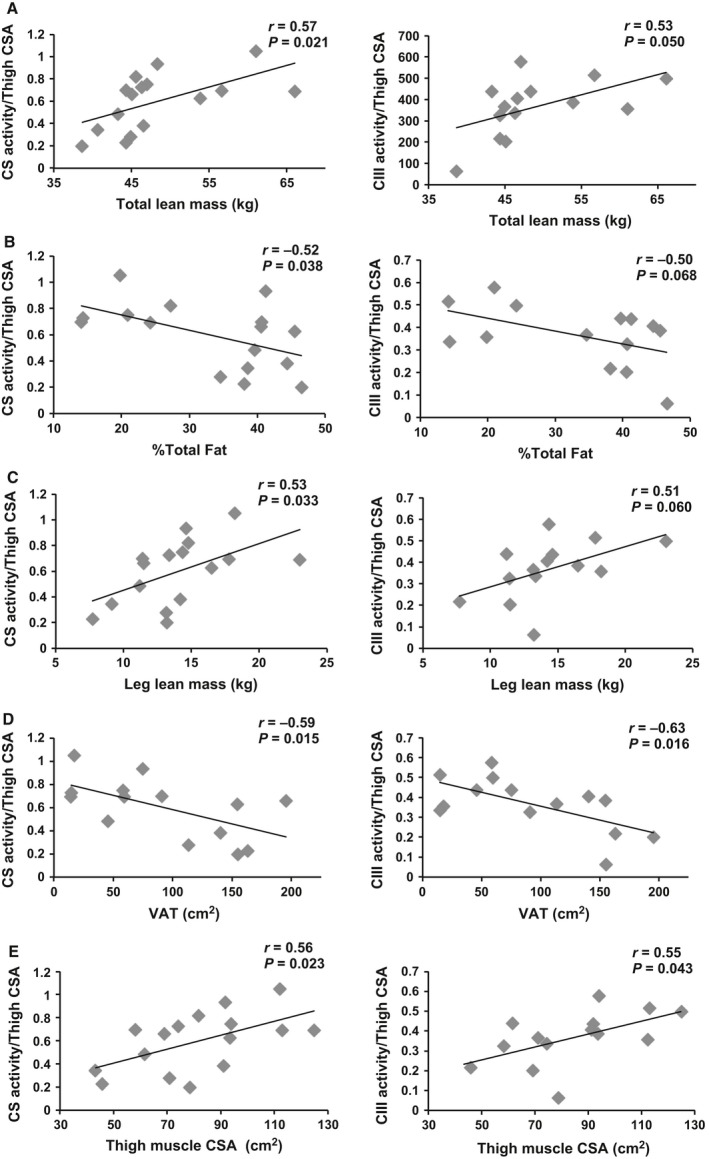
CS and antimycin A‐sensitive complex III (CIII) activity normalized to midthigh CSA related to body composition measurements including total lean mass (A), %total fat (B), leg lean mass (C), thigh muscle CSA (D), and VAT (E). (A–C) *n* = 18 for CS,* n* = 15 for CIII, (D) *n* = 16 for CS,* n* = 14 for CIII, and (E) *n* = 17 for CS,* n* = 14 for CIII. CS, Citrate synthase; CSA, cross‐sectional area; VAT, visceral adipose tissue.

Importantly, CS normalized to thigh muscle CSA was related to total lean mass (*r* = 0.57, *P* = 0.021), %total body fat (*r* = −0.52, *P* = 0.038), and leg lean mass (*r* = 0.53, *P* = 0.033; Figure 4A–C). CIII normalized to thigh muscle CSA was related to total lean mass (*r* = 0.53, *P* = 0.050) with a trend for significance between %total fat (*r* = A−0.50, *P* = 0.068) and leg lean mass (*r* = −0.51, *P* = 0.060; Figure 4A–C).

### Relationships among mitochondrial mass, activity, VAT, and SAT

VAT (*r* = −0.66, *P* = 0.0038) and the ratio of VAT/SAT (*r* = −0.62, *P* = 0.008) were negatively related to CS activity (*n* = 17, Fig. 2E). Similarly, there was a negative correlation between VAT and CIII activity (*r* = −0.60, *P* = 0.022), but not with VAT/SAT ratio (*r* = −0.45, *P* = 0.10; Fig. 3E). Finally, SAT was not related to CS or CIII activity (data not shown). These relationships were no longer statistically significant when age was accounted for; however, there was a trend for a relationship between CS and VAT independent of age (Table 3). Partial correlations to account for TSI revealed significant relationships between CS and VAT with a trend for significance between CIII and VAT (Table 3). When corrected for thigh muscle CSA, CS (*r* = −0.59, *P* = 0.015) and CIII (*r* = −0.63, *P* = 0.016) were related to VAT (Fig. 4D).

### Relationships among mitochondrial mass, activity, and skeletal muscle

**Figure 5:**
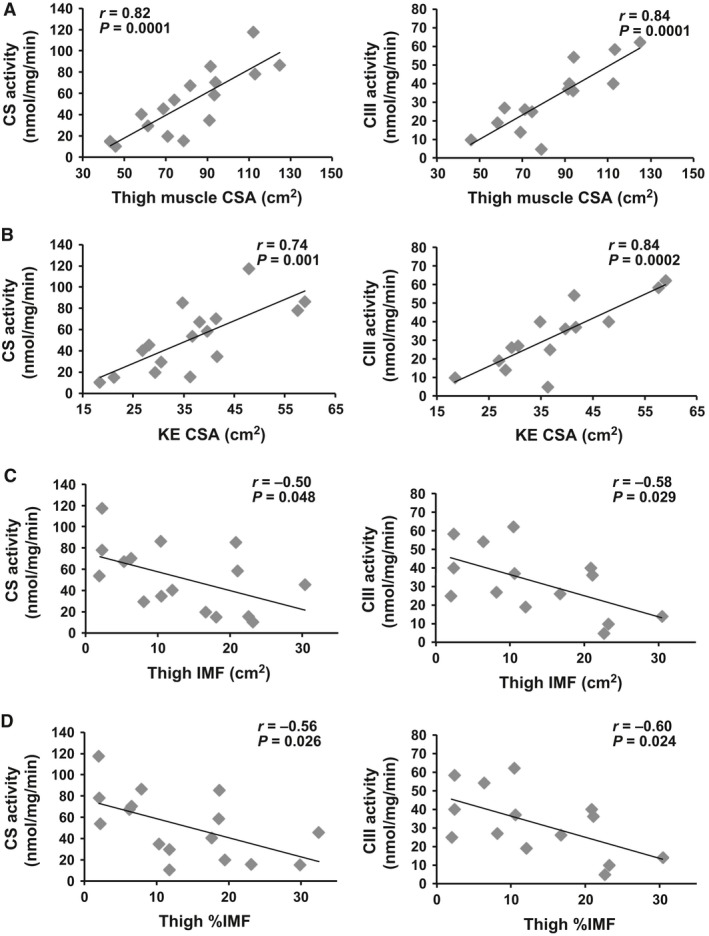
Citrate synthase (CS;* n* = 16) and antimycin A‐sensitive complex III (CIII;* n* = 14) activity related to thigh muscle cross‐sectional area (CSA; A), knee extensor (KE) CSA (B), thigh intramuscular fat (IMF; C), and thigh %IMF (D).

The relationship between CS or CIII activity and thigh skeletal muscle is shown in Figure 5 (*n* = 16 for CS and *n* = 14 for CIII). Thigh analysis was excluded for one participant because of a large heterotopic ossification that interfered with analysis of muscle cross‐sectional area. There was a positive correlation between CS activity and thigh muscle CSA (*r* = 0.82, *P* = 0.0001) or knee extensor CSA (*r* = 0.74, *P* = 0.001). Similarly, there was a positive relationship between CIII activity and thigh muscle CSA (*r* = 0.84, *P* = 0.0001) or knee extensor CSA (*r* = 0.84, *P* = 0.0002). These relationships remained statistically significant when age or TSI was accounted for (Table 3). Furthermore, CS (*r* = 0.56, *P* = 0.023) and CIII (*r* = 0.55, *P* = 0.043) normalized to thigh muscle CSA were positively related to thigh muscle CSA (Fig. 4E).

Total thigh IMF was negatively related to both CS (*r* = −0.50, *P* = 0.048) and CIII activity (*r* = −0.58, *P* = 0.029). Percentage IMF in the midthigh was negatively related to CS (*r* = −0.56, *P* = 0.026) and CIII (*r* = −0.60, *P* = 0.024). These relationships were no longer statistically significant when age was accounted for. Interestingly, when TSI was accounted for the relationship between CIII and %thigh IMF remained (Table 3). Knee extensor IMF was not related to CS or CIII activity (data not shown). CIII activity normalized to CS activity was not correlated with any body composition measurements (*P* > 0.05).

### Mitochondrial mass and activity after SCI

**Figure 6:**
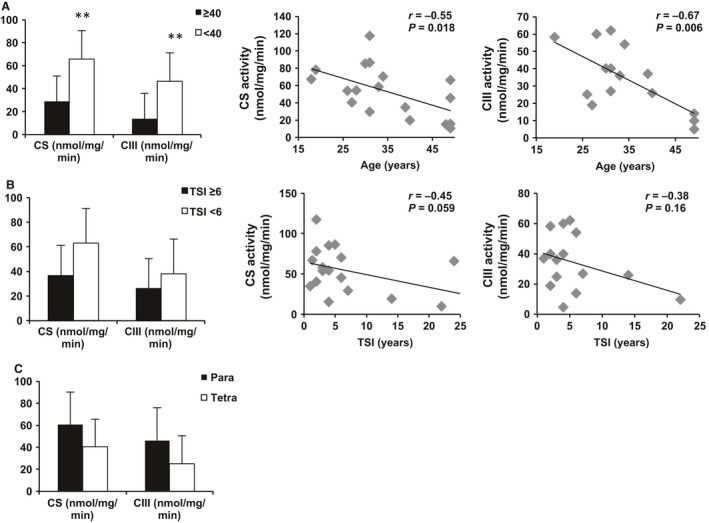
Differences in citrate synthase (CS) and antimycin A‐sensitive complex III (CIII) activity between older (≥40; *n* = 6 for CS,* n* = 4 for CIII) and younger (<40; *n* = 12 for CS,* n* = 11 for CIII) individuals with SCI and the correlation with CS and CIII activity (A). CS and CIII activity in individuals with time since injury (TSI) greater than (*n* = 7 for CS,* n* = 5 for CIII) or less than 6 years (*n* = 11 for CS,* n* = 10 for CIII) and correlations are shown in (B). Differences between CS and CIII activity in paraplegics (para; *n* = 11 for CS,* n* = 9 for CIII) and tetraplegics (tetra; *n* = 7 for CS,* n* = 6 for CIII) are shown in (C). **P < 0.001. SCI, Spinal cord injury.

Figure 6 describes the difference in CS and CIII activity between groups. There was significantly increased CS (*P* = 0.009) and CIII (*P* = 0.004) activity in younger individuals with SCI (<40) compared with older (≥40). Similarly, there was a negative relationship between age and CS (*r* = −0.55, *P* = 0.018) and CIII (*r* = −0.67, *P* = 0.006).

There was a trend for decreased CS activity (*P* = 0.06) in individuals who were injured over ≥6 years ago but no change in CIII activity (*P* = 0.25). There was a trend for a negative relationship between CS and TSI (*r* = −0.45, *P* = 0.059) and between CIII and TSI (*r* = −0.38, *P* = 0.16). Similarly, individuals with tetraplegia had slightly lower CS (*P* = 0.16) and CIII (*P* = 0.12) activity compared to individuals with paraplegia. There was a slight but nonsignificant negative relationship between level of injury and CS (*r* = −0.35, *P* = 0.16) and CIII (*r* = −0.42, *P* = 0.12; data not shown). There was no relationship between ethnicity or BMI (>25 vs. <25) and CS or CIII activity.

## Discussion

This study demonstrates that adipose tissue deposition is negatively related to skeletal muscle mitochondrial mass and CIII activity in individuals with chronic SCI. In contrast, lean mass and thigh skeletal muscle CSA are positively related to mitochondrial enzyme activities. Additionally, our observation that VAT and IMF are negatively related to mitochondrial enzyme activities suggests that ectopic adipose tissue may negatively influence mitochondrial health. Finally, the present findings suggest that advanced age, but not level of injury or TSI, is associated with decreased mitochondrial enzyme activity in skeletal muscle from individuals with SCI.

Individuals with SCI experience severe muscle atrophy and changes in body composition that increase the risk of developing metabolic disorders such as type II diabetes, insulin resistance, and cardiovascular disease (Elder et al. 2004; Gorgey et al. 2014; Jensen 2008; Spungen et al. 2000). Previous studies have suggested a decrease in skeletal muscle mitochondrial activity after SCI (Erickson et al. 2013; Grimby et al. 1976; Martin et al. 1992; McCully et al. 2011). However, little is known about the relationship between mitochondrial activity and body composition after SCI (O'Brien and Gorgey 2016). In this study, mitochondrial health was determined by CS and CIII activity, surrogate markers for mitochondrial mass and ETC activity, respectively.

Complex III was chosen for analysis because it is decreased in diseases including peripheral artery disease (Brass et al. 2001) and neurodegenerative diseases (Itoh et al. 2013). Furthermore, complexes I and III are sites of free radical leakage from the ETC and are vulnerable to injury by free radicals (Turrens 2003). Our findings are similar to those observed in a previous study with individuals with peripheral artery disease (Brass et al. 2001). Per unit CS, the skeletal muscle CIII activity measured in this study (0.75 ± 0.13; *n* = 16) was similar to that of individuals with peripheral artery disease (0.95 ± 0.18; *n* = 17) and approximately 50% of control subjects (1.47 ± 0.33; *n* = 9)(Brass et al. 2001).

### Mitochondrial mass, activity, and body composition

This study found that as %fat increases, CS and CIII activity decreases in individuals with SCI. The opposite was true for total lean mass and thigh muscle CSA. This is consistent with a previous report that skeletal muscle CS activity was negatively related to %fat, total fat mass, and BMI in healthy, able‐bodied individuals over age 65 (Bharadwaj et al. 2015). Previous studies have also showed negative relationships between regional body composition and metabolic profile (Lalia et al. 2016). CIII activity normalized to CS activity was not correlated with body composition measurements. This suggests that mitochondrial mass, not CIII activity per mitochondrion, is driving the relationship.

The mechanism underlying the relationship between an increase in adipose tissue or muscle mass and mitochondrial activity remains unclear. However, recent studies suggest that substrate utilization may play a role. Substrates can enter the ETC through CI or CII. Maximal skeletal muscle respiration through both complexes I and II was found to be negatively related to %fat (Lalia et al. 2016) and BMI (Distefano et al. 2016). However, another study found no relationship between maximal complex II respiration and %fat, total lean mass, or thigh muscle CSA in able‐bodied individuals (Bharadwaj et al. 2015).

When age was accounted for, measures of total and leg lean mass, but not %fat, remained correlated with CS and CIII. Similarly, Bharadwaj et al. (2015) found that %fat and thigh muscle CSA but not total lean mass were related to CS when BMI, gender, and age were accounted for. Interestingly, when TSI was accounted for, measures of total and regional fat and lean mass remained significantly related to mitochondrial enzyme activities. Our data also show that CS and CIII activity are related to body composition measurements even when normalized to thigh muscle CSA. Collectively, these data suggest that increasing muscle mass and/or decreasing %fat mass may improve mitochondrial mass independent of age or TSI. Taken together, the current findings support a relationship between body composition and mitochondrial enzyme activities in SCI. This suggests that individuals with more lean mass have more mitochondria and that inducing skeletal muscle hypertrophy may be an effective rehabilitation tool to increase skeletal muscle mitochondrial activity.

### Mitochondrial mass, activity, VAT, and SAT

Another main finding of this study is that mitochondrial enzyme activities related differently based on the distribution of adipose tissue. Android fat mass but not leg or trunk fat mass was negatively related to CIII activity with a trend for CS. Furthermore, VAT, but not SAT, was negatively related to both CS and CIII activity. Previously, we have shown that VAT, but not SAT, negatively influences metabolic profile in individuals with SCI. In these studies, SAT distribution served as a protective parameter for high‐density lipoprotein cholesterol and glucose utilization (Gorgey and Gater 2011b; Gorgey et al. 2011).

The relationship between VAT and mitochondrial mass was independent of age and TSI, providing credence to the significance of exploring interventions in order to reduce VAT after SCI. These findings are also in line with a study that found that individuals with increased abdominal obesity had decreased skeletal muscle mitochondrial ATP production and ATP‐to‐oxygen ratio (Chanseaume et al. 2010). However, these data are in contrast to a recent study that found no relationship between skeletal muscle mitochondrial respiration and VAT or SAT (Lalia et al. 2016). The reason for these discrepant results remains unclear; however, the method of analyzing mitochondrial activity was different in each study. Chanseaume et al. (2010) measured respiration of permeabilized muscle fibers, whereas Lalia et al. (2016) isolated mitochondria. This study measured mitochondrial enzyme activity in tissue homogenates, not isolated mitochondria.

Respiration is currently the gold standard for assessing mitochondrial respiratory chain function; however, the process of isolating mitochondria has been suggested to alter metabolic activity (Picard et al. 2010). For this reason, analysis of isolated skeletal muscle mitochondria may not be as representative of physiological function as intact muscle fibers. Because of the need to freeze biopsy samples, this study measured enzymatic activity instead of mitochondrial respiration. While we did not directly measure mitochondrial function, CS activity has been shown to be related to mitochondrial content measured by transmission electron microscopy and muscle oxidative capacity measured by respiration (Larsen et al. 2012).

Another ectopic adipose tissue type similar to VAT, IMF, is seen with muscle disuse and has been associated with increased insulin resistance (Elder et al. 2004; Goodpaster 2013; Pellegrinelli et al. 2015). IMF was negatively related to both CS and CIII activities in this study. This is in agreement with studies that found that IMF was negatively related to CS (Bharadwaj et al. 2015; Simoneau et al. 1995). However, a recent study found no relationship between IMF and maximal CII‐driven respiration (Bharadwaj et al. 2015). Furthermore, a recent publication found no relationship between mitochondrial respiration and intramyocellular lipids measured by proton magnetic resonance spectroscopy in the tibialis anterior of healthy subjects (Lalia et al. 2016). These discrepant findings may be a result of studying different muscles and using different methods of analysis.

### Mitochondrial mass and activity after SCI

Advanced age predisposed individuals with SCI to decreased mitochondrial health. There was a significant decrease in CS and CIII activity in individuals with SCI over age 40 compared with those under age 40, consistent with investigations in able‐bodied individuals (Chanseaume et al. 2010; Lalia et al. 2016; Short et al. 2005, 2003). Other studies found a negative relationship between CS activity and age (Short et al. 2005, 2003). Complex I, complex II, and complex IV activities were negatively related to age as well (Short et al. 2005, 2003). Similarly, recent studies reported decreased mitochondrial respiration in older individuals compared to young active individuals (Chanseaume et al. 2010) and a negative relationship between respiration and age (Lalia et al. 2016). This study extends these findings to men with chronic SCI. However, our current data only suggest relationships, not causality. In order to build on our findings, future studies could test directional hypotheses in order to shed light on these complicated dynamics.

### Limitations

Due to limited sample size, only CS and CIII enzyme activities were analyzed. Linked CI/CIII activity was assessed in 10 samples by NADH‐cytochrome c oxidoreductase activity as described previously (Brass et al. 2001); however, the activity was either undetectable or so low that it was unreliable (data not shown). Because NADH dehydrogenase is thought to be the rate‐limiting step in this assay, these findings may suggest a decrease in complex I activity in the SCI population. Further research comparing muscle samples from SCI and able‐bodied individuals would be needed in order to determine this.

Creatine kinase, a marker of muscle fiber content, was not measured in this study. While every effort was made to remove adipose tissue and connective tissue from the muscle sample, a small amount may remain. However, the citrate synthase values reported here are similar to previous studies that analyzed human skeletal muscle tissue homogenates (MacInnis et al. 2016; Simoneau and Kelley 1997). Future studies will measure creatine kinase in order to normalize data to muscle fiber content. Although the CS values presented here are similar to those previously published, there is a wide range. This could be due to differences in study populations. For example, a recent study published maximal CS values within 1.8 mmol/kg per min even after exercise training; however, this study was conducted on 10 active men aged 22–24 (MacInnis et al. 2016). Another study observed a larger range (>2 fold) in CS values between lean and obese nondiabetic subjects and those with non‐insulin‐dependent diabetes mellitus, however, these subjects only ranged in age from 44 to 54 (Simoneau and Kelley 1997). This study included individuals ranging in age from 18 to 50 with levels of injury ranging from C5 to T11, and TSI between 1 and 28 years.

Individual ETC enzyme activities represent maximum activity and one limitation of this type of analysis is that it may not represent physiological activity of the ETC Future research will be aimed at examining the amount of free radical leakage from the ETC in skeletal muscle from SCI individuals, as well as functional assays of mitochondrial activity (i.e., respiration). Other, noninvasive measures of mitochondrial activity need to be explored as well, such as near‐infrared spectroscopy.

Another limitation of this study is that only males participated, and all had complete injuries. However, it is worth nothing that men represent 80% of the SCI population. Current studies are aimed at recruiting both men and women and those with incomplete as well as complete injuries.

## Conclusion

In summary, this study has demonstrated for the first time relationships among body composition, visceral adiposity, skeletal muscle, and mitochondrial mass and activity in individuals with SCI. These findings support the use of interventions that increase lean mass and decrease adipose tissue, particularly around the waist. These interventions may prove beneficial to individuals regardless of age or TSI. An increase in mitochondrial mass will increase cellular energy production that may result in an increase in energy expenditure and may decrease comorbidities in chronic SCI and other populations.

## Conflict of Interest

The authors declare no conflict of interest.
